# Editorial: Digital brain health

**DOI:** 10.3389/fdgth.2023.1142897

**Published:** 2023-02-14

**Authors:** Lars Lau Raket, Paula Petcu, Katarzyna Wac, Jason Hassenstab

**Affiliations:** ^1^ Novo Nordisk, Copenhagen, Denmark; ^2^ Brain+, Copenhagen, Denmark; ^3^ Quality of Life Technologies Lab, University of Geneva, Geneva, Switzerland; ^4^ Department of Neurology, Washington University in St. Louis, St. Louis, MO, United States

**Keywords:** digital health, neuroscience, remote assessment, cognition, brain health

**Editorial on the Research Topic** Digital brain health

Digital health technology has the potential to revolutionize how we monitor brain health and diagnose, monitor, and treat brain diseases by providing a range of innovative tools and services. These technologies include wearable and external devices, mobile apps, and telemedicine platforms.

While diagnosis, disease monitoring and intervention are traditionally addressed using distinctly separate tools, these different use cases may be overlapping or integrated in single solutions in the digital brain health space. For example, sleep trackers can simultaneously monitor sleep quality, give suggestions for improving sleep and potentially detect changes in patterns that could be early signs of brain disorders like depression or Alzheimer's disease.

The articles in this research topic on digital brain health explore novel approaches to using technology in relation to brain health, and consider important questions related to implementation and utility of such solutions. In the present topic, all articles focus on the ability for digital health technologies to deliver remote solutions. Most of the articles study remote assessment of cognition and symptoms through mobile devices, but there are also examples of cognitive and electrophysiological biomarker assessments using at-home equipment and a study of how brain-computer interfaces can enable better social integration of people with motor disabilities.

In total, this topic on digital brain health contains seven papers that are briefly summarized below.

Nicosia et al. explore stigmas and challenges in relation to digital brain health studies in older adults. This question is particularly relevant since older adults are simultaneously the ones most at risk of cognitive decline and least familiar with technology. Results demonstrate that while older age was indeed associated with less technological familiarity, the majority of older adults that were offered to participate elected to participate in the smartphone-based study and those participating showed exceptional adherence.

Berron et al. report a study on feasibility of completely unsupervised digital cognitive assessments through a mobile app in a Citizen Science project including 1,407 adults aged 18–89 across Germany. Their study suggests that fully unsupervised remote memory assessments are feasible, but also identified critical factors that may influence both compliance and performance that should be considered in future studies.

Öhman et al. report a study of the validity of unsupervised mobile app-based cognitive testing in a Swedish 1944 birth cohort (aged 76–77 at time of study) of non-demented participants. They found that mobile app-based cognitive scores on individual tasks were weakly-to-moderately correlated with conventional cognitive tests and that single-session data showed poor-to-moderate test-retest reliability. When using the average of two sessions, test-retest reliability improved substantially.

Elbin et al. review motivations for using remote assessments in concussion clinical care and present data from a pilot study using smartphone-based ambulatory assessments to capture patient reports of symptom severity, environmental exposures, and performance-based assessments of cognition.

Barbey et al. report encouraging results in relation to the feasibility and quality of repeated, at-home, self-administered wireless dry EEG to measure brain function.

Brugada-Ramentol et al. present perspectives of the use of virtual reality systems for cognitive training and monitoring and present the design of a specific immersive virtual reality system intended to achieve these tasks.

Finally, Lazarou et al. report a study on the usability of a brain-computer interfaces in people with motor disabilities to better support the use of multimedia, including social media. Such technology can help support social integration of people with movement disorders, and the study generally reported favorable evaluations of how the platform helped users to achieve better social integration.

